# Evaluation of Liver Fibrosis Using Shear Wave Elastography after Surgery for Congenital Biliary Dilatation

**DOI:** 10.24546/0100492148

**Published:** 2024-11-21

**Authors:** YUDAI TSURUNO, HIROAKI FUKUZAWA, MITSUMASA OKAMOTO, TOSHIFUMI TADA

**Affiliations:** 1 Department of Pediatric Surgery, Japanese Red Cross Society Himeji Hospital, Himeji, Japan; 2 Department of Internal Medicine, Japanese Red Cross Society Himeji Hospital, Himeji, Japan; 3 Department of Pediatric Surgery, Research Field in Medical and Health Sciences, Medical and Dental Area, Research and Education Assembly, Kagoshima University, Kagoshima, Japan; 4 Department of Pediatric Surgery, Medical Research institute Kitano Hospital, PIIF Tazuke-kofukai, Osaka, Japan

**Keywords:** Congenital biliary dilatation, Liver fibrosis, Shear wave elastography, Postoperative, Complications

## Abstract

**PURPOSE:**

Liver fibrosis is not thought to occur in patients with no adverse events after surgery for congenital biliary dilatation (CBD). However, this speculation is not supported by any reports. Real-time shear wave elastography (SWE) is a noninvasive, ultrasound-based technique to evaluate liver stiffness. We aimed to clarify the presence of liver fibrosis using SWE in patients who had undergone surgery for CBD.

**METHODS:**

We included patients who underwent radical surgery for CBD, who were followed up until March 2022, and have been performed with SWE at our institution from April 2021 to March 2022. Liver stiffness was evaluated using SWE, and liver fibrosis stages (F0–F4; METAVIR scoring) were determined based on the previously reported associations between liver stiffness and liver fibrosis. We assessed the general condition of each patient and performed routine blood investigations on the same day as SWE.

**RESULTS:**

Two out of 20 patients had long-term complications (intrahepatic stones without symptoms [n = 1], recurrent cholangitis [n = 1]). The median hepatic shear wave propagation velocity was 1.26 (range, 1.12–1.60) m/s in all cases. The estimated liver fibrosis stage was ≤F1 in patients without long-term complications. In patient with recurrent cholangitis, the hepatic shear wave propagation velocity was 1.60 m/s, and the estimated liver fibrosis stage was F3.

**CONCLUSION:**

Liver fibrosis tended not to occur in patients with no complications after surgery for CBD. However, patients with long-term postoperative complications, such as cholangitis, should be examined using SWE so as not to overlook liver cirrhosis.

## INTRODUCTION

Congenital biliary dilatation (CBD) is associated with pancreaticobiliary maljunction. The standard surgical procedure for CBD is complete resection of the extrahepatic bile duct and biliary reconstruction, during which Roux-en-Y hepaticojejunostomy is usually performed. The postoperative period is uneventful in most patients, and, unlike biliary atresia, liver fibrosis is not thought to occur in cases without postoperative adverse events. However, there is no report confirming the absence of liver fibrosis in such patients because liver biopsy, an invasive procedure, is not usually performed in these cases.

Recent advancements have produced alternative noninvasive methods for estimating the degree of liver fibrosis [1–3]. Real-time shear wave elastography (SWE) has been developed as a noninvasive, ultrasound-based technique to evaluate liver stiffness. SWE measures the velocity of the shear wave emitted from the echo probe as it propagates through the tissue. This velocity is directly related to the stiffness of the tissue, called the modulus of elasticity (E = 3 qv^2^, where v is the shear rate and q is the density of the tissue). The stiffer the tissue, the faster the shear wave propagates [4]. This method can easily and accurately assess the degree of liver fibrosis in clinical practice [5–8].

SWE has already been used to assess biliary atresia in pediatric patients. Chen et al. established the usefulness of SWE in predicting liver fibrosis in patients who underwent surgery for biliary atresia [9]. Leschied et al. reported that SWE could help diagnose biliary atresia [10]. However, SWE has not yet been used to evaluate liver fibrosis in patients with CBD. This study aimed to clarify the presence of liver fibrosis using SWE in patients who had undergone surgery for CBD.

## MATERIALS AND METHODS

### Methods

The study was approved by our institutional review board (2021–27). It complies with the Health Insurance Portability and Accountability Act. Informed consent was obtained from patients over 16 years of age or their legal guardians for patients aged <16 years. Participants in this study were patients who underwent radical surgery for CBD, who were followed up until March 2022, and have been performed with SWE at our institution from April 2021 to March 2022. All patients underwent radical surgery, which involved complete excision of the extrahepatic bile duct and Roux-en-Y hepaticojejunostomy. We evaluated the liver stiffness of each patient during the postoperative follow-up. SWE was performed without sedation by ultrasound technicians who were blinded to the patients’ clinical data. Moreover, we assessed each patient’s general condition and performed routine blood investigations during the same visit. Continuous variables are expressed as median (range) values.

### Shear wave elastography image acquisition and analysis

**Figure 1:**
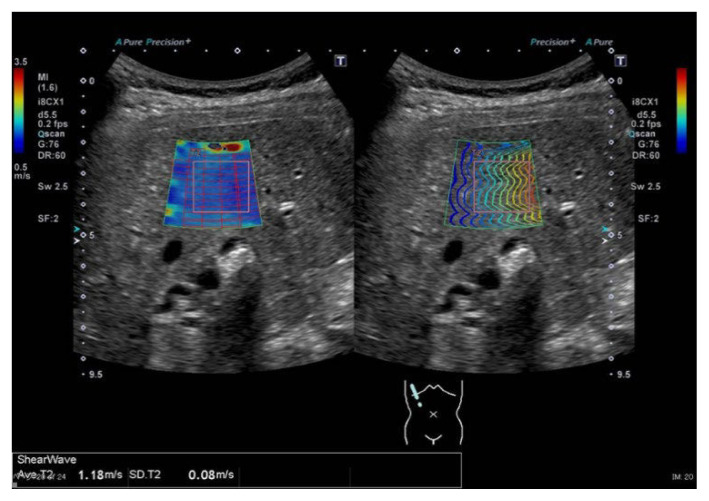
Measurement of liver stiffness using shear wave elastography

SWE imaging was performed in all patients in the outpatient ultrasonography room. SWE measurements were performed using an Aplio-500 or Aplio-i800 device and a 375BT probe (Canon Medical Systems, Tokyo, Japan). During the examination, the patients were placed in the supine position. The tip of the transducer probe was placed between the ribs over the right lobe of the liver. The detection site was fixed 1.0 to 2.0 cm beneath the surface of the right liver, away from the intrahepatic vessels. Three successful acquisitions at different locations were obtained for each patient, the mean value was calculated, and the results were expressed as shear wave propagation velocity (m/s) (Figure 1).

In order to estimate the degree of liver fibrosis, we used the data on the correlation of liver stiffness with liver fibrosis from the report by Iijima et al. [11]. They reported a comparison of liver stiffness measured using the same device of the “Aplio series” (Canon Medical Systems, Tokyo, Japan) and stages of liver fibrosis based on liver biopsy findings. This report indicated that the respective median liver stiffness values were 1.36, 1.54, 1.67, and 2.42 m/s in patients with liver fibrosis stages F1, F2, F3, and F4 (the METAVIR scoring system) [12]. In general, liver fibrosis is considered clinically significant when it spreads beyond the portal tract (F2, F3, or F4) and clinically non-significant when it is absent or restricted to the portal tract (F0 or F1) [13]. The stages of liver fibrosis in our patients were estimated in comparison with these data.

## RESULTS

### Patient characteristics

Patient characteristics are described in Table I. Twenty patients (male, n = 6; female, n = 14) underwent SWE during the study period. Excluding one case with no records, the median age at the time of radical surgery was 3.33 (range, 0.6–14.2) years, and the median follow-up period was 9.45 (range, 2.97–24.19) years. The median age at SWE assessment was 15.84 (range, 7.21–27.18) years. Based on the Todani classification [14], five cases were classified as Ia, five as Ic, seven as IV-A, and three as unknown. The blood test results on the same day as SWE were normal in almost all cases, with the exception of a few clinically irrelevant variations in some patients (Table I).

**Figure 2:**
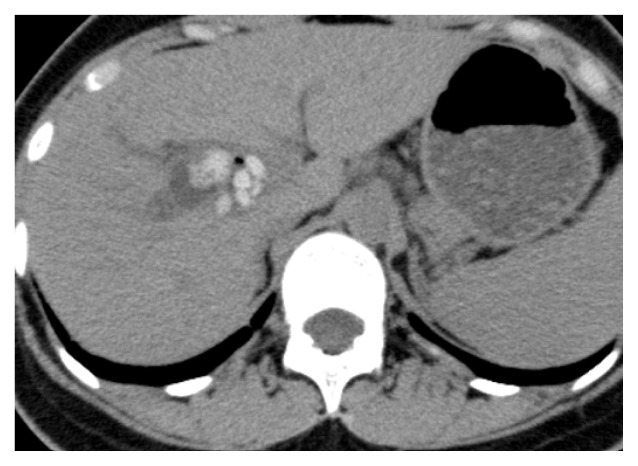
Multiple intrahepatic stones in the right hepatic duct were detected by CT scan.

Long-term complications were identified in two patients. One patient with Todani IV-A disease underwent radical surgery at 1 year of age. Multiple intrahepatic stones were detected in the right hepatic duct during postoperative follow-up examinations (Figure 2). However, the patient did not have any symptoms and had normal blood test results for 25 years. The patient deferred treatment as she was asymptomatic. Therefore, only observation has been continued until now.

**Figure 3:**
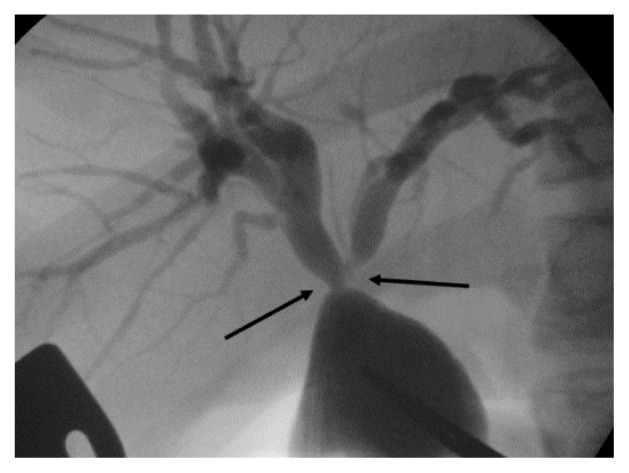
Stenosis of hilar hepatic duct (black arrow) was confirmed on intraoperative cholangiography during re-operation.

Another patient with Todani IV-A disease underwent radical surgery at 2 years of age. After the surgery, repeated recurrence of cholangitis was observed and treated with antibiotics in each instance. The condition persisted for 10 years. Subsequently, hilar hepatic duct stenosis was detected by DIC-CT. Therefore, hepatic ductoplasty and re-anastomosis were performed when the patient was 12 years old (Figure 3). The cholangitis resolved after the operation, and blood test results were normal. The patient has since recovered, and cholangitis has not recurred as of the most recent follow-up.

### Liver stiffness and estimation of liver fibrosis

The median hepatic shear wave propagation velocity was 1.26 (range, 1.21–1.60) m/s in all cases. In all but two patients with long-term complications, median hepatic shear wave propagation velocity was 1.25 (range, 1.21–1.41) m/s. The estimated liver fibrosis stage in all patients without long-term complications was F0 in 12 cases and F1 in 6 cases, suggesting that the degree of liver fibrosis was clinically irrelevant.

Conversely, the hepatic shear wave propagation velocity at the examination 8 years after re-operation in the patient with recurrent cholangitis was 1.60 m/s, and the estimated liver fibrosis stage was F3. In another patient with long-term complications (asymptomatic intrahepatic stones), the hepatic shear wave propagation velocity at the examination 24 years after operation was 1.21 m/s, and the estimated liver fibrosis stage was F0, indicating the absence of liver fibrosis (Table II).

## DISCUSSION

In this study, we used SWE to investigate the occurrence of liver fibrosis in patients without long-term complications after surgery for CBD.

SWE has recently become very popular as a noninvasive technique for investigating liver fibrosis in adults. Moreover, SWE is widely used to examine other organs, such as the mammary glands. In the pediatric field, it is often used for the diagnosis and postoperative follow-up of biliary atresia, and its utility has been established. However, there have been no reports on the usefulness of SWE in patients with CBD.

Liver fibrosis has not been considered to occur in patients with no long-term complications after surgery for CBD. However, there are no reports that support this assumption. The present study is the first to confirm that liver fibrosis does not occur in such patients. Although the number of included patients is not very large, the SWE data obtained are uniform. Therefore, we believe that it is a reliable sample of patients without complications after surgery for CBD.

The measurement of liver stiffness using SWE can be affected by various inflammatory diseases of the liver. Specifically, it has been reported that when the alanine aminotransferase (ALT) levels in the blood increase, shear wave velocity also increases [15]. However, in the present study, the blood ALT levels were normal in all patients. Therefore, the measured liver stiffness was considered to be purely related to the stage of liver fibrosis.

In the present study, the age at which SWE was performed ranged from 7.21 to 27.18 years. The liver stiffness of healthy children has been discussed in the literature [16–19]. Cailloce et al. measured the liver stiffness in a normal child using the same device used in the present study (Aplio i800, Canon). The median hepatic shear wave propagation velocity was 1.225 m/s [19]. The results were almost identical to that obtained in our study, suggesting that liver fibrosis does not occur in patients with no adverse events after surgery for CBD.

In the present study, clinical evidence of liver fibrosis was only noted in one patient with a 10-year history of recurrent cholangitis. Clinical symptoms disappeared after reoperation, and the blood test results were normal. This patient’s good condition is maintained for more than 9 years. However, liver fibrosis persisted. In contrast, the patient with intrahepatic stones had no history of cholangitis. Likewise, they did not have liver fibrosis. These data suggest that repeated cholangitis, rather than intrahepatic stones, is strongly involved in the pathogenesis of liver fibrosis.

Cholangitis can be a direct cause of liver fibrosis after surgery for CBD. Thus, it is important to determine and manage the cause in patients with postoperative recurrent cholangitis. In patients who undergo surgery for CBD, long-term postoperative complications, such as cholangitis, should be examined using SWE to avoid potential liver cirrhosis.

SWE can be measured using devices from various manufacturers. It has been reported that there is almost no difference between the data obtained with devices of varying models or from different manufacturers [11]. Therefore, in the future, it will be possible to use SWE to measure liver stiffness postoperatively in patients who undergo surgery for CBD, and the results can be verified despite the use of various models at multiple centers.

## LIMITATIONS

In the present study, the METAVIR scoring system was used to assess liver fibrosis. However, this scoring system is based on data from patients with hepatitis C, and we could not verify whether postoperative liver fibrosis and fibrosis caused by viral hepatitis were similar. Furthermore, in this study, the number of patients is small and follow-up periods is not long enough. More patients and longer follow-up are needed.

## CONCLUSION

As we had expected, we found that liver fibrosis tended not to occur in patients with no complications after surgery for CBD. However, patients with long-term postoperative complications, such as cholangitis, should be examined using SWE so as not to overlook liver cirrhosis.

## Figures and Tables

**Table I:** Characteristics and blood test results of patients on the day of Shear Wave Elastography evaluation

Variable (N = 20)	Value
Sex	
Male (n)	6
Female (n)	14
Age at operation years, median (range)	3.33 (0.6–14.2)
Postoperative period, years, median (range)	9.45 (2.97–24.19)
Age at shear wave elastography, years, median (range)	15.84 (7.21–27.18)
Todani classification	
Ia (n)	5
Ic (n)	5
IV-A (n)	7
Unknown (n)	3
Aspartate aminotransferase, IU/L, median (range)	19 (16–30)
Alanine aminotransferase, IU/L, median (range)	15 (9–30)
Gamma-glutamyl transpeptidase, IU/L, median (range)	12 (6–27)
Total bilirubin, mg/dL, median (range)	0.6 (0.3–2.4)
Direct bilirubin, mg/dL, median (range)	0.1 (0.0–0.5)
Alkaline phosphatase, IU/L, median (range)	264 (139–1,125)
Long-term complications (n)	2
Intrahepatic calculus without any symptoms (n)	1
Recurrent cholangitis (n)	1

**Table II:** Liver stiffness and liver fibrosis

Variable	Value
Median (range) hepatic shear wave propagation velocity of all patients (m/s)	1.26 (1.12–1.60) (n = 20)
Median (range) hepatic shear wave propagation velocity of patients without long-term complications (m/s)	1.25 (1.21–1.41) (n = 18)
Hepatic shear wave propagation velocity of patients with long-term complications (m/s)	1.60 (recurrent cholangitis 1.21 (asymptomatic intra-hepatic stones)
Estimated liver fibrosis stage (n)	
Without long-term complications (18)	F0 (12), F1 (6)
With long-term complications (2)	F0 (1), F3 (1)
